# Progesterone Gel and Placebo Prolonged Pregnancy More Effectively Than Intravenous Tocolysis Alone in Women with Preterm Labor

**DOI:** 10.3390/gels8050272

**Published:** 2022-04-26

**Authors:** Ylva Vladic Stjernholm, Tomislav Vladic, Giovanna Marchini

**Affiliations:** 1 Department of Women’s and Children’s Health, Karolinska University Hospital and Karolinska Institutet, Akademiska Stråket 14, 171 64 Stockholm, Sweden; tomislav.vladic@telia.com; 2 Neonatal Unit, Astrid Lindgren’s Children’s Hospital, Eugeniavägen 23, 171 64 Solna, Sweden; giovanna.marchini@regionstockholm.se

**Keywords:** cervical ripening, microbiome vaginal, preterm birth, preterm labor, progesterone, tocolytics

## Abstract

The aim of this trial was to evaluate the effect of progesterone gel compared to placebo in prolonging pregnancy among women with preterm labor. **Methods:** A randomized controlled trial in Sweden in 2009–18. Women with early preterm labor were randomized to daily doses of progesterone gel 90 mg (n = 28) or placebo (n = 30) after standard intravenous tocolytics. Women with intravenous tocolytics alone (n = 29) served as controls. **Results:** The median latency to delivery was 68 (range 28–88) days with progesterone and 72 (range 9–90) days with placebo (*p* = 0.84), compared to 1 (range 1–2) day in the control group (progesterone and placebo vs. control *p* < 0.001). The rate of preterm birth before 34 weeks was 32% after progesterone and 37 % after placebo (*p* = 0.32) compared to 100 % in the control group (*p* < 0.001, respectively). The composite neonatal morbidity (*p* = 0.65) and neonatal intensive care unit admission (*p* = 0.12) were comparable between the progesterone and placebo groups and lower in these groups compared with neonates in the control group (*p* < 0.001, respectively). **Conclusions:** Progesterone gel and placebo were equally effective in prolonging pregnancy among women with early preterm labor, and both treatments were more effective than standard intravenous tocolysis alone. We hypothesize that the acidic placebo gel reinforced the biochemical barrier at the uterine cervix, which counteracts ascending pathogen invasion and subsequent inflammation, and thereby prevented preterm labor.

## 1. Introduction

The global rate of preterm birth (PTB)—the main cause of neonatal, infant and child mortality up to 5 years of age—is still 10% [1]. Risk factors include psychosocial stress, malnutrition, low and high maternal age, multiple pregnancy, decidual bleeding, ascending pathogen invasion of the amniotic sac and uterus, and alterations in the vaginal microbiome [2,3,4,5,6,7,8]. Current tocolytic treatments do not prevent PTB, but are given with an aim to delay delivery for at least 48 h to optimize the effect of antenatal corticosteroids for fetal lung maturation and allow for transport to a tertiary hospital with Neonatal Intensive Care Unit (NICU) expertise [9]. A cervical length (CL) ≤ 25 mm in early pregnancy is regarded as a primary predictor for PTB [10].

The connective tissue remodeling of the uterine cervix that precedes term and preterm labor is characterized by an increased density of macrophages, release of proinflammatory cytokines and prostaglandin E, a functional progesterone withdrawal, activation of metalloproteinase (MMP) enzymes, a changed proteoglycan composition with dispersion of collagen fibrils, and collagen degradation. These biochemical events lead to cervical effacement and dilatation that allow for childbirth [4,5,6,7,8,11].

Progesterone is regarded as the primary hormone for pregnancy maintenance, but reports on prophylactic treatment with bioidentical progesterone or synthetic progestins such as 17α-hydroxyprogesterone caproate (17OH−PC) for the prevention of PTB are inconclusive [3]. Bioidentical progesterone has no androgenic effects that might affect the lipid metabolism or harm the fetus. Since oral progesterone is poorly absorbed because of the liver metabolism, daily progesterone injections would be painful, a transdermal progesterone preparation has not been available in obstetrics, vaginal treatment is used in clinical practice [12,13]. Prophylactic treatment, which is recommended by the International Federation of Gynecology and Obstetrics (FIGO) for asymptomatic women with a previous PTB or a short CL, is effective according to some studies except those with the largest sample size [3,13]. Extensive screening programs are needed to evaluate prophylactic strategies since only 10–20% of women with spontaneous PTB have a previous PTB [2,4] and only 1–2% of asymptomatic women have a CL ≤ 25 mm in early pregnancy [10,14].

The aim of this study was to compare the effect of progesterone gel and placebo in prolonging pregnancy among women with early preterm labor (PTL). The treatments started after standard intravenous tocolytics. We hypothesized that progesterone would be more effective than the placebo [15]. Women who received intravenous tocolytics alone served as a control group.

## 2. Materials and Methods

This single center trial was conducted at the Obstetric Unit, Department of Women’s and Children’s Health, Karolinska University Hospital and Karolinska Institutet, Stockholm, Sweden between 2009–18.

Ethics approval was obtained from the Regional Ethics Board for Medical Sciences in Stockholm and registered 05/09/2007, No. 2007-311-31. The trial was registered at the European Union Drug Regulating Authorities Clinical Trials (EudraCT) that participates in the World Health Organization’s (WHO) International Clinical Trial Registry Platform, registered 17/12/2007, registration No. 2007-003348-31, and was approved by the Swedish Medical Products Agency on 15/05/2008, registration number 151:2008/30388. All treatments were performed according to the relevant clinical guidelines and regulations, and all participants and parents of participants below 16 years were included after informed oral and written consent. Ethics approval for the control group of women, who received standard intravenous tocolytics alone in 2009–18, was obtained from the Regional Ethics Board for Medical Sciences in Stockholm on 09/04/2015, registration No. 2014/255-31. Since data from the control group were collected in retrospect and presented on a group basis only, individual informed consent from participants in the control group was not required from the Regional Ethics Board for Medical Sciences in Stockholm.

**Figure 1:**
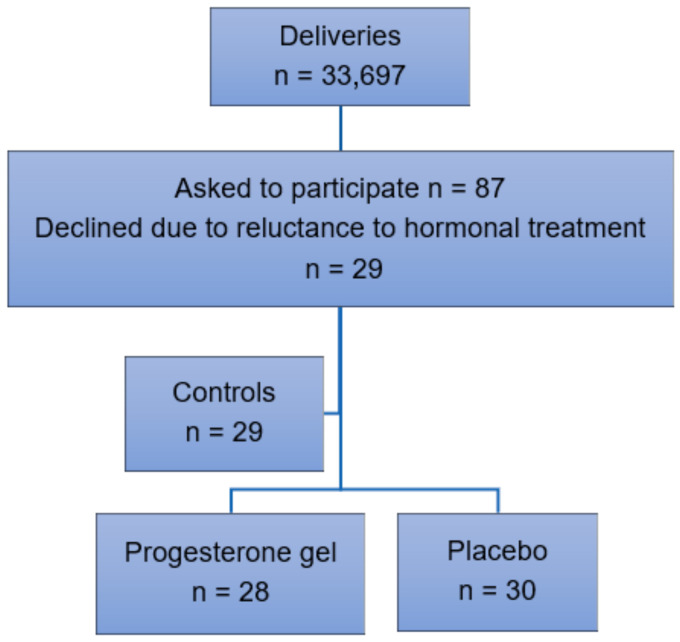
Inclusion of participants in 2009–18.

**Participant recruitment.** Inclusion criteria were singleton pregnancy, intact fetal membranes, and early spontaneous PTL between 24–28 gestational weeks resulting in a CL < 25 mm as determined by transvaginal ultrasound. Exclusion criteria were multiple pregnancy, ruptured fetal membranes, cervical dilatation, cervical cerclage, signs or symptoms of chorioamnionitis, previous uterine surgery, prophylactic progesterone treatment, intercurrent maternal disease, pregnancy complications such as preeclampsia or gestational diabetes, intrauterine fetal growth restriction, or fetal malformations (Figure 1). Oral and written information about the trial was provided by an obstetrician at the hospital. Randomization using a standard computerized system was carried out after oral and written consent. Data were collected in retrospect from a control group of women with identical inclusion and exclusion criteria who received treatment with standard intravenous tocolytics alone in 2009–18. Preterm labor was uterine contractions ≥ 2/10 min for > 30 min according to cardiotocography (CTG) recorded in electronic obstetric records (Obstetrix, Cerner AB, Stockholm, Sweden), which resulted in a CL < 25 mm as determined by transvaginal ultrasound carried out by a specialist in obstetrics and gynecology due to standardized criteria.

**Interventions.** The progesterone group received daily vaginal progesterone gel (Crinone, 90 mg/dose, Merck KGaA, Gernsheim, Germany). The placebo group received placebo gel (Replens, CampusPharma AB, Göteborg, Sweden), an emulsion of oil and water with an acidic pH of 3.0. Unfortunately, it was impossible to blind the gel packages at the pharmacy. The treatments were given after standard intravenous tocolytics and continued until 34 + 0 weeks, rupture of the fetal membranes, or childbirth—whatever occurred first. In the years studied, intravenous tocolytics consisted of a bolus dose of the oxytocin receptor antagonist atosiban (Tractocile, Ferring Pharmaceuticals, Limhamn, Sweden) 6.75 mg followed by infusion of 300 μg/min during 3 h and thereafter 100 μg/min until 48 h. Alternatively, a β2-adrenergic receptor agonist terbutaline (Bricanyl, AstraZeneca PLC, Luton, UK) 5 μg/mL was given for 48 h according to the individual obstetrician’s choice. All women received two doses of betamethasone (Betapred, Swedish Orphan Biovitrum AB, Solna, Sweden) 12 mg intramuscularly 12–24 h apart for fetal lung maturation. According to clinical guidelines, intrapartum prophylaxis with bensylpenicillin 3 g every 6 h was given to delivering women in active labor < 37 + 0 weeks. Women in all groups were seen weekly by an obstetrician after discharge from the hospital in case of regression of PTL.

**Outcomes.** The analyses included all randomized participants according to the intention to treat concept. We did not expect dropouts, since the limited group of participants in this single center study was randomized after thorough informed consent and were seen frequently by an obstetrician. The primary outcome latency to delivery was calculated from the first gel dose to childbirth in the treatment groups and from the start of standard intravenous tocolysis to childbirth in the control group. The secondary outcomes were delivery ≤ 7 days, rates of PTB < 34 weeks and < 37 weeks, neonatal birth weight (BW), composite neonatal morbidity, NICU admission, and length of NICU stay. Composite neonatal morbidity was Apgar score < 7 at 5 min, the incidence of neonatal respiratory distress syndrome (RDS), intraventricular hemorrhage (IVH), necrotizing enterocolitis (NEC), and sepsis ≤ 7 days, taken together with retinopathy of prematurity (ROP) and neonatal death during NICU stay. Neonatal RDS was defined by clinical diagnosis of type I RDS and a requirement of oxygen therapy for at least 24 h. Maternal adverse effects such as fatigue, headache, or intrahepatic cholestasis were monitored.

**Sample size.** We hypothesized, according to clinical observations taken together with results from a previous report [15], that the latency to delivery would be 35 days with progesterone compared to 7 days with placebo. According to a power analysis, a sample size of (n = 29) in each group would be required to reach a significance of 5% and power of 80% [16].

**Statistical analysis.** Continuous data were analyzed using the Mann–Whitney U-test and were presented as mean ± standard deviation (SD) or median and interquartile range. Categorical data were analyzed with Chi^2^-test and Fisher’s exact test when appropriate and were presented as numbers and percentages. Confidence intervals and composite neonatal morbidity were analyzed with one-way ANOVA. A two-tailed *p* value < 0.05 was considered significant.

## 3. Results

We evaluated the effect of vaginal progesterone gel (n = 28) compared to placebo (n = 30) in preventing PTB among women with early PTL, see Figure 1. In the years 2009–18, n = 33,697 childbirths took place at our hospital. In total, n = 87 women were asked to participate in the trial. Of these, n = 29 women declined due to reluctance to hormonal treatment during pregnancy. This group of women with identical inclusion and exclusion criteria received standard intravenous tocolytics alone and served as a control group.

**Maternal characteristics.** Maternal characteristics are shown in Table 1. The demographic data were comparable between the groups. The participants in all groups had a medical history of a previous PTB in 25% and first or second trimester spontaneous abortion in 25% (data not shown). The median gestational age (GA) at treatment start was 26 (25–27) weeks in the progesterone group, 26 (25–27) weeks in the placebo group, and 26 (25–28) weeks at intravenous tocolytics start in the control group. The CL (mean ± SD) at treatment start was 11 ± 5 mm in the progesterone group, 12 ± 5 mm in the placebo group, and 14 ± 7 mm in the control group. The mean circulating level of the inflammatory marker C-reactive protein (CRP) was low, ≤10 mg/L, in the groups (data not shown).

**Maternal outcome.** The maternal outcome is shown in Table 2. The latency to delivery (median and interquartile range, IQR) was 68 (28–88) days in the progesterone group and 72 (9–90) days in the placebo group (*p* = 0.84). The rate of PTB < 34 weeks was 32% with progesterone and 37% with placebo (*p* = 0.32), and the rate of PTB < 37 weeks was 54% with progesterone and 43% with placebo (*p* = 0.65). The compliance rates were high. One participant in each group had her treatment interrupted before 34 weeks after referral to another hospital, and both gave birth after 37 weeks. One woman in the progesterone group, who had an emergency cervical cerclage on maternal request after inclusion, continued her progesterone treatment and gave birth at 34 weeks. No severe maternal side effects such as headache or intrahepatic cholestasis were reported. One woman in the progesterone group reported fatigue. In the control group, the latency to delivery was 1 (1–2) day (progesterone and placebo vs. control *p* < 0.001), and the rate of PTB < 34 weeks was 100% (progesterone vs. control *p* = 0.01; placebo vs. control *p* = 0.02).

**Neonatal outcome.** The neonatal outcome is shown in Table 3. The neonatal BW (median and IQR) was 2700 (1202–3215) g in the progesterone group and 2506 (1252–3310) g in the placebo group (*p* = 0.84). The individual rate of composite neonatal morbidity was 0.75 in the progesterone group and 0.63 in the placebo group (*p* = 0.65). The rate of NICU admission was 32% (9/28) after progesterone and 23% (7/30) after placebo, and the median length of NICU stay was 0 (0–10) days in both groups (*p* = 0.59). Two neonatal deaths occurred during the NICU stay in the progesterone group and one in the placebo group due to postnatally diagnosed severe malformations and chromosomal aberrations. Neonates in the control group had a BW of 934 (730–1050) g (progesterone and placebo vs. control *p* < 0.001), an individual composite morbidity rate of 1.96 (progesterone and placebo vs. control *p* < 0.001), a 100 % (29/29) rate of NICU admission and a median NICU stay of 75 (43–107) days (progesterone and placebo vs. control *p* < 0.001). Two neonatal deaths occurred during the NICU stay.

## 4. Discussion

We have compared the effect of maintenance treatment with vaginal progesterone gel to placebo in prolonging pregnancy among women with early PTL. The treatments were given after standard intravenous tocolytics, and women with identical inclusion and exclusion criteria who received intravenous tocolytics alone served as controls.

The results showed that progesterone and placebo were equally effective in prolonging pregnancy, and the hypothesis was therefore rejected. The primary outcome, latency to delivery, and the secondary outcomes, rate of delivery < 7 days and rates of early and late PTB < 34 and < 37 weeks, were comparable. As a consequence, neonates in the progesterone and placebo groups had comparable mean BW, composite neonatal morbidities, rates of NICU admission, and lengths of NICU stay. However, both progesterone and placebo were more effective in prolonging pregnancy than standard intravenous tocolytics alone. Women in progesterone and placebo groups had longer latency to delivery, lower rates of delivery < 7 days, and lower rates of early and late PTB than women in the control group. Neonates in the treatment groups had higher mean BW, lower composite morbidities, lower rates of NICU admission, and shorter lengths of NICU stay compared to controls.

The present results suggest that the acidic placebo gel with a pH of 3.0 was effective in prolonging pregnancy, most likely by reinforcing the biochemical barrier at the uterine cervix, which counteracts ascending pathogen invasion and subsequent inflammation, known triggers of PTL. A physiological *Lactobacillus*-dominated vaginal microbiome promotes an acidic pH < 4.5 and constitutes a biochemical barrier against ascending pathogens from the skin and bowel microbiota to the uterus. During pregnancy, the vaginal microbiome undergoes significant changes resulting in an even lower pH than in the non-pregnant state. In contrast, alterations in the vaginal microbiome resulting in a higher pH are associated with PTB [7,17]. The present results are in accordance with reports on uterine infection due to ascending pathogen invasion as an etiology behind 30–60% of all early PTB [2]. The present findings are also in agreement with reports showing that the acidic placebo gel exerts anti-inflammatory effects, decreases the cervical collagen-metabolizing enzyme MMP-13, and delays PTB [18].

Randomized trials on maintenance treatment with vaginal progesterone after the onset of PTL are warranted, as only a few trials on this topic have been published [19]. Two trials report longer latency to delivery after vaginal progesterone compared to no treatment [15,20], one trial reports longer latency to delivery after progesterone compared to placebo [21], and one reports no differences after vaginal progesterone, intramuscular progestin 17OH-PC, or no treatment [22]. The diverse results between the trials could be explained by the different study designs. Participants in the present trial were included at a median GA of 26 weeks in contrast to 31 weeks or more [15,20,21] and ≥ 28 weeks [22] in earlier studies. Spontaneous PTL was ≥ 2 uterine contractions per 10 min for > 30 min resulting in a CL < 25 mm without cervical dilatation determined by transvaginal ultrasound in the present trial and one of the previous trials [22], in contrast to cervical shortening and/or softening or dilatation [15], cervical softening, shortening at least by 50% and dilatation < 2 cm [20], or cervical dilatation and/or effacement [21] assessed by digital examination. Transvaginal ultrasound is recommended for CL determination in women with PTL, and the accuracy of digital assessment is questioned [10,13,14]. Intravenous tocolysis in the present trial consisted of an oxytocin receptor antagonist or a β2-receptor agonist in contrast to magnesium sulfate in combination with ampicillin [15], a β2-receptor agonist [20], magnesium sulfate in combination with pethidine and ampicillin [21], an oxytocin receptor antagonist, a calcium blocker, or a non-steroidal anti-inflammatory drug (NSAID) [22]. The maintenance treatment consisted of progesterone gel 90 mg in the present trial, in contrast to vaginal progesterone suppositories 400 mg [15] or 200 mg [20,21,22] in previous reports.

Different bioavailabilities of the progesterone preparations could have influenced the results. However, the clinical effects are similar when vaginal progesterone gel is compared to progesterone suppository for luteal phase support in early pregnancy [23].

Strengths of this trial were the consistent inclusion and exclusion criteria, the transvaginal ultrasound CL determination, and that all data were retrieved from original electronic obstetric records at one hospital. Limitations were the lack of a double-blinded design and the slow inclusion of participants. It is possible also that participation in the progesterone and placebo groups in itself reduced chronic psychosocial stress, which is a known risk factor of PTL, and thereby prolonged pregnancy [24,25].

## 5. Conclusions

The present results showed that vaginal progesterone gel and placebo were equally effective in prolonging pregnancy among women with early PTL and that both treatments were more effective than standard intravenous tocolysis alone. We hypothesize that the acidic placebo gel reinforced the biochemical barrier at the uterine cervix, which counteracts ascending pathogen invasion and subsequent inflammation, known risk factors for PTL. Our results suggest that non-hormonal agents such as the acidic placebo gel could be useful for the prevention of PTB in clinical practice.

## Figures and Tables

**Table 1:** Maternal characteristics.

Variable	Progesterone	Placebo	Control
	n = 28	n = 30	n = 29
Age, years, mean ± SD	31 ± 4	29 ± 6	32 ± 5
BMI, kg/m^2^, mean ± SD	24 ± 5	23 ± 3	24 ± 2
Primiparous, n (%)	12 (43)	16 (53)	16 (55)
GA, weeks, median (IQR)	26 (25–27)	26 (25–27)	26 (25–28)
CL, mm, mean ± SD	11 ± 5	12 ± 5	14 ± 7
Tocolysis, atosiban/terbutaline, n (%)	24/4 (86/14)	26/4 (87/13)	26/3 (90/10)

Abbreviations: BMI = Body Mass Index; CL = Cervical Length; GA = Gestational Age; IQR = Interquartile Range, n = number of participants.

**Table 2:** Maternal outcome. Statistical methods: Mann–Whitney U-test, general linear model and one-way ANOVA^1^, and Chi^2^-test and Fisher’s exact test^2^.

Variable	Progesterone n = 28	Placebo n = 30	*p* Value PR vs. PL	Control n = 29	*p* Value PR vs. C	*p* Value PL vs. C
Latency, d median (IQR)	68 (28–88)	72 (9–90)	0.84^1^	1 (1–2)	< 0.001^1^	<0.001^1^
Delivery ≤ 7 d n (%)	4 (14)	6 (20)	0.06^2^	29 (100)	< 0.001^2^	<0.001^2^
PTB < 34 + 0 wks n (%)	9 (32)	11 (37)	0.32^2^	29 (100)	0.01^2^	0.02^2^
PTB < 37 + 0 wks n (%)	15 (54)	13 (43)	0.65^2^	29 (100)	0.06^2^	0.04^2^

Abbreviations: C = Control; d = days; Delivery < 7 d = delivery < 7 d after treatment start; IQR = Interquartile Range; Latency = Latency from treatment start to childbirth; n = number of participants; PTB = Preterm Birth; PL = Placebo; PR = Progesterone, wks = weeks.

**Table 3:** Neonatal outcome. Statistical methods: Mann–Whitney U-test and one-way ANOVA^1^; Chi^2^-test and Fisher’s exact test^2^.

Variable	Progesterone	Placebo	*p* Value	Control	*p* Value	*p* Value
	n = 28	n = 30	PR vs. PL	n = 29	PR vs. C	PL vs. C
BW, g, median (IQR)	2700 (1202─3215)	2506 (1252─3310)	0.84^1^	934 (730–1050)	<0.001^1^	<0.001^1^
Composite morbidity, n (IR)	21 (0.75)	19 (0.63)	0.65^2^	57 (1.96)	<0.001^2^	<0.001^2^
Apgar < 7 at 5 min	3	2		9		
IVH	1	2		4		
NEC	2	1		2		
RDS	8	8		27		
Sepsis	3	4		8		
ROP	2	1		5		
Death	2	1		2		
NICU admission, n (%)	9 (32)	7 (23)	0.12^2^	29 (100)	<0.001^2^	<0.001^2^
NICU, d, median (IQR)	0 (0–10)	0 (0–10)	0.59^1^	75 (43–107)	<0.001^1^	<0.001^1^

Abbreviations: BW = Birth Weight; C = Control; Composite morbidity = Apgar < 7 at 5 min, IVH (Intraventricular Hemorrhage), NEC (Necrotizing Enterocolitis), RDS (Respiratory Distress Syndrome), and sepsis ≤ 7 days, taken together with ROP (Retinopathy of Prematurity) and death during NICU stay; IR = Individual Rate; IQR = Interquartile Range; d = days; n = number of participants; NICU = Neonatal Intensive Care Unit; PL = Placebo; PR = Progesterone.

## Data Availability

Data from this study will be provided by the authors if requested.

## Abbreviations

BW, Birth Weight; CL, Cervical Length; IVH, Intraventricular Hemorrhage; IQR, Interquartile Range; MMP, Matrix Metalloproteinase Enzyme; NEC, Necrotizing Enterocolitis; NICU, Neonatal Intensive Care Unit; PTB, Preterm Birth, PTL, Preterm Labor, RDS, Respiratory Distress Syndrome; ROP, Retinopathy of Prematurity.
